# A Novel Rapid 3D Tissue-Clearing and Staining Approach for Enteric Neurovascular Imaging and Pathology Applications

**DOI:** 10.3390/diagnostics16050759

**Published:** 2026-03-03

**Authors:** Debao Li, Xuqing Cao, Jienan Lin, Qingchi Zhang, Rui Dong, Song Sun, Chun Shen

**Affiliations:** 1 National Health Commission (NHC) Key Laboratory of Neonatal Diseases, Department of Pediatric Surgery, Children’s Hospital of Fudan University, Shanghai 201102, China; lidebaoemail888@163.com (D.L.); 22111240002@m.fudan.edu.cn (X.C.); rdong@fudan.edu.cn (R.D.); 2 Department of Neonatal Surgery, The Affiliated Women and Children’ s Hospital of Ningbo University, Ningbo 315012, China; felinjienan@nbu.edu.cn; 3 Xiamen Key Laboratory of Pediatric General Surgery Diseases, Children’s Hospital of Fudan University (Xiamen Branch), Xiamen Children’s Hospital, Xiamen 361006, China; zqc1403@126.com

**Keywords:** neurovascular imaging, intestinal pathologies, 3D staining and imaging, tissue clearing, confocal microscopy

## Abstract

**Background and Aims:** Neurovascular abnormalities, such as aberrant nerve migration in Hirschsprung’s disease and reduced vascular density in necrotizing enterocolitis, are frequently observed in intestinal diseases. Traditional 2-dimensional (2D) staining methods are complicated, time-consuming and fail to comprehensively visualize the intricate neurovascular structures and morphology of the intestine. This study focuses on evaluating a novel 3D staining technique that promises simpler, faster, and more effective visualization of intact neurovascular structures in the colon. Additionally, it aims to compare the strengths and limitations of this 3D method against traditional 2D techniques for analyzing neuronal and vascular changes in two prevalent pathological conditions. **Methods:** A novel tissue-clearing approach was used to render mouse and patient distal colon tissues transparent. Neural structures and blood vessels were stained. 2D and 3D imaging were performed with laser confocal or tiling light sheet microscopy. Parameters include total imaging time, imaging range, image quality, operational complexity, and post-processing were compared between 2D and 3D methods. **Results:** Compared to 2D imaging, 3D imaging reveals the complete morphology and trajectory of neurovascular structures. Confocal 3D imaging offers superior clarity, higher transparency, and faster workflow efficiency, whereas light-sheet microscopy provides broader coverage at the expense of lower image quality. Post-processing facilitated spatial modeling and quantitative analyses. Applications included Hirschsprung’s disease, where 3D imaging revealed abnormal nerve distribution, and congenital heart disease, where hypoperfusion impacted vascular development in the colon. **Conclusions:** Confocal 3D staining and imaging offered a more streamlined workflow and enabled comprehensive visualization of neurovascular architecture, supporting efficient assessment of intestinal neurovascular phenotypic features.

## 1. Introduction

To effectively maintain normal absorption and digestive functions, the intestine possesses a hierarchically branched vascular network as well as a sophisticated, autonomous enteric nervous system, which is often referred to as the “second brain” [1]. The development of the neurovascular system in the murine intestine begins at gestational day 9.5, continues throughout the entire gestation period, and extends to the later stages of postnatal life [2,3]. A well-organized, branched vascular network nourishes the functional cells of the intestine, facilitating nutrient absorption, and the intricate enteric nervous system regulates intestinal motility as well as secretion, blood flow, and immune responses. However, even minor disturbances to the neurovascular system during gestation or at birth can have fatal consequences for infants, such as Hirschsprung disease (HSCR) resulting from abnormal neural migration or necrotizing enterocolitis (NEC) in premature infants caused by localized intestinal ischemia [4,5,6].

The microscopic visualization of neurovascular structures in the intestine, both under normal and pathological conditions, is crucial for understanding disease pathophysiology, as well as for accurate diagnosis and effective treatment strategies. To examine these structures, researchers typically employ two-dimensional (2D) slicing techniques for staining and observation. Common methods include hematoxylin and eosin staining for subcellular structural marking, as well as immunohistochemistry and in situ hybridization for molecular labeling, followed by optical or electron microscopy imaging. Although 2D staining techniques demonstrate satisfactory performance in terms of tissue resolution and antibody labeling efficiency, significant limitations persist. First, sectioning provides only a cross-sectional view of the tissue’s microarchitecture, failing to capture the overall trajectory and morphology of neurovascular structures, resulting in considerable loss of sample information. Second, artifacts and distortions caused by the sectioning process can negatively impact subsequent observations and experimental outcomes. Lastly, the intricate tissue processing procedures and the precise angular requirements for sectioning pose substantial challenges for researchers. Collectively, these limitations severely restrict our ability to observe neurovascular structures at the microanatomical level.

Recent advancements in tissue clearing and three-dimensional (3D) imaging techniques offer promising solutions to the limitations inherent in traditional 2D sectioning methods for visualizing neurovascular structures [7,8]. Tissue clearing involves the removal of lipids and other light-scattering substances from biological tissues, rendering them transparent without significant structural loss [9]. This process enhances light penetration, allowing for clearer imaging of internal structures. By facilitating the visualization of intact neurovascular structures, tissue-clearing techniques enhance our understanding of neurovascular architecture, offering a more comprehensive view than conventional methods [10].

3D imaging is based on the principle of creating a series of optical sections by adjusting the focal plane at various depths within the sample, ultimately reconstructing a comprehensive 3D image. To achieve effective 3D imaging, laser confocal microscopy and light sheet microscopy are frequently employed, each offering distinct advantages. Confocal microscopy excels in capturing high-resolution images with enhanced contrast due to its ability to exclude out-of-focus light, making it particularly suitable for detailed structural analyses [11]. Conversely, light sheet microscopy minimizes photobleaching and phototoxicity, allowing for high-speed imaging, which is especially beneficial for observing dynamic biological processes in living specimens [12,13]. This adaptability in imaging techniques significantly enhances our understanding of neurovascular structures and their roles in various intestinal pathologies [14,15].

In this study, we achieved efficient and high-quality imaging of intact neurovascular structures in the colon using novel tissue clearing and 3D staining techniques [16]. We utilized anti-Neuron-Specific Class III Beta-Tubulin (β3-tubulin) and anti-vasoactive intestinal peptide (VIP) antibodies for 3D staining of the neural components and marked blood vessels with wheat germ agglutinin (WGA) dye. 3D imaging was performed using either laser confocal microscopy or light sheet microscopy. We compared 3D imaging with conventional 2D imaging across several parameters, including total workflow time (from tissue clearing to staining and imaging), field of view, image quality, operational difficulty, and post-processing ability. Additionally, we will discuss the applications of tissue clearing and 3D staining techniques in two common clinical conditions: Hirschsprung’s disease and systemic blood hypoperfusion.

## 2. Methods

### 2.1. Study Design

The experiment included colon tissue samples from 60 mice and 15 children with Hirschsprung’s disease (HSCR). WT mice were purchased from Jihui (Jihui Biotechnology, Shanghai, China), and *Ednrb*^−/−^ mice were provided by Dr. Rui Dong’s lab. All mice were housed in a 19–22 °C SPF environment with ad libitum access to food and water under a 12-h light/dark cycle. Two lactating female mice were housed together in a single cage to nurse the pups until tissue collection on postnatal day 10. The congenital systemic hypoperfusion model was generated by pulmonary artery banding (PAB) for neonatal mice following the protocols described in our previously published studies [17,18]. A total of 60 mice were divided into two groups: a nerve imaging group and a vascular imaging group, with 30 mice in each group. Within each group, the mice were further divided into three subgroups based on imaging modality: 10 mice for 2D imaging, 10 for 3D confocal imaging, and 10 for light-sheet microscopy. For each imaging modality subgroup, 5 mice were assigned to the control group and 5 to the experimental group (HSCR or systemic hypoperfusion model). The grouping for vascular staining experiments followed the same structure. For human HSCR specimens, narrowed segments and dilated segments were obtained from 15 patients (a total of 30 samples) from January 2024 to December 2024. Each group of 10 specimens (from 5 patients) was subjected to 2D staining, 3D confocal imaging, and 3D light-sheet microscopy for subsequent analysis. Clinical characteristics of the enrolled patients and the usage-based subgroup comparison are summarized in Supplementary Tables S1 and S2.

All animal experimental procedures were approved by the Institutional Animal Care and Use Committee (IACUC) of Fudan University Children’s Hospital. All human experiments were approved by the ethical review boards from the Ethics Committee of the Pediatric Hospital Affiliated to Fudan University. All procedures followed the Declaration of Helsinki for human participants and the Animal Welfare Act for animal studies. The study is reported in accordance with ARRIVE guidelines. Mice were anesthetized with isoflurane and euthanized by administering 100 mg/kg body weight of pentobarbital sodium via intraperitoneal injection. Clinical specimens were obtained during routine medical procedures with informed consent from parents or legal guardian of the patients.

### 2.2. WGA Perfusion and Sample Preparation

**Figure 1:**
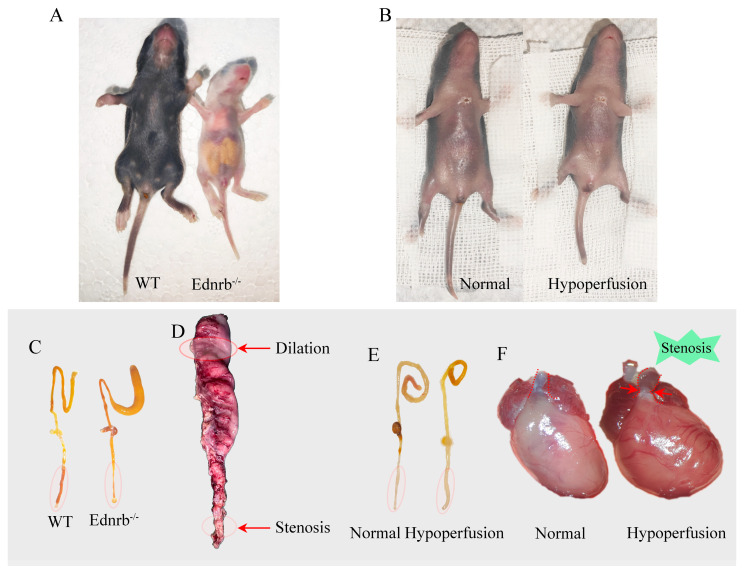
Gross images of tissues and organs in HSCR and reduced systemic perfusion models. (**A**) Gross images of P10 HSCR (*Ednrb*^−/−^) transgenic mice and normal mice, showing apparent abdominal distension in HSCR transgenic mice, resembling clinical presentation. (**B**) Gross images of P10 control and systemic hypoperfusion (PAB) mice. (**C**) Gross images of intestines from normal and *Ednrb*^−/−^ mice, displaying distinct dilated and narrowed segments. (**D**) Gross images of human colonic tissues from HSCR patients, with red circles indicating the narrowed and dilated segments. (**E**) Gross images of colonic tissues from normal and reduced systemic perfusion groups, with red circles marking the segments used in the experiment. (**F**) Gross images of hearts from P10 normal and reduced systemic perfusion groups, with red dashed lines and arrows indicating the modeling site at the narrowed right ventricular outflow tract.

WGA (W32466, Thermo Fisher Scientific Inc., Waltham, MA, USA) was administered via a 20 µL apical injection of a 1 mg/mL solution into the left ventricle, confirmed by the presence of blood return in the syringe. To prevent coagulation, sodium heparin was injected intraperitoneally, and the mice were allowed to move freely for 5 min. Then mice were anesthetized with isoflurane. After confirming the absence of reflexes through the toe pinch, a V-shaped incision was made to open the thoracic cavity. A small incision was created at the right atrium. In total, 10 mL phosphate-buffered saline (PBS, Beyotime Biotechnology, Shanghai, China) followed by 10 mL 4% paraformaldehyde (PFA, Beyotime Biotechnology, Shanghai, China) were used for systemic perfusion through the apex at a controlled rate of 1 mL/min. A perfusion pump was used to ensure stable perfusion speed. The perfusion procedure facilitates the removal of circulating red blood cells, which is crucial for enhancing the clearing effect and improving tissue transparency [19]. The effectiveness of perfusion can be confirmed by observing whether the liver changes from red to white (Figure 1B). Upon completion of the perfusion, tissue samples from the proximal colon to the rectum were collected, followed by the removal of intestinal contents and surrounding connective tissues. Gross imaging of the mouse and samples was conducted using a stereomicroscope (Leica Microsystems, Wetzlar, Germany) (Figure 1A–C). Gross photographs of human HSCR colon tissues were captured using a camera (Figure 1D). In the systemic hypoperfusion group, the colon and the hearts were also excised and photographed to validate the modeling efficacy (Figure 1E,F). Pathological and control bowel segments were excised using sterile instruments. Segments were identified intraoperatively by the pediatric surgeon and verified by intraoperative rapid frozen-section immunohistochemistry; postoperative formal histology further confirmed the resection margins. Dilated segments were sampled at the site of maximal diameter. Tissue samples were fixed (24 h for 2D and confocal-3D preparations, and 24–48 h for light-sheet 3D) at 4 °C and then washed three times with PBS to remove any residual fixation solution, preparing them for subsequent use. To minimize experimental variability introduced by the WGA perfusion procedure, samples were excluded from analysis when any of the following criteria were met: (i) Unexplained perfusion failure, indicated by an absence of vascular fluorescence., (ii) death within 5 min post-perfusion, and (iii) gross thoracic hemorrhage noted at harvest.

### 2.3. 2D Staining and Imaging

For frozen sections, tissues were dehydrated overnight in a 30% sucrose solution (Beyotime Bio. Shanghai, China) until they completely settled at the bottom. The tissues were then embedded in OCT (Sakura Finetek Japan Co., Ltd., Tokyo, Japan) compound within molds, rapidly frozen in liquid nitrogen, and stored at −80 °C. After freezing overnight, the tissues were sliced into 10 µm thick sections. For paraffin sections, tissues were fixed in 4% PFA and then processed through a series of ethanol solutions for dehydration. Following this, the tissues were cleared with xylene and embedded in paraffin. Thin sections, 5–10 µm in thickness, were prepared using a microtome. The sections were washed three times and outlined with an oil-based marker to prevent water from spreading. They were then incubated in a blocking solution (PBS containing 7.5% goat serum and 0.5% Triton X-100, Beyotime Biotechnology, Shanghai, China) for 2 h.

The sections were then stained with anti-β3-tubulin (TUJ1, ab78078, Abcam, Cambridge, UK) and anti-VIP (ab272726, Abcam, Cambridge, UK) antibodies to label the nerves and CD31(ab182981, Abcam, Cambridge, UK) to label the vessel. The samples were incubated at 4 °C overnight for 16 h. The following day, the sections were brought to room temperature for 30 min and washed three times with PBST (PBS containing 0.1% Tween, ST1726, Beyotime Biotechnology, Shanghai, China). Next, they were incubated with secondary antibodies: either Anti-rabbit IgG (H+L), F(ab’)2 Fragment (Alexa Fluor^®^ 555 Conjugate, 4413S, Cell Signaling Technology, Boston, MA, USA; dilution: 1:500) or Anti-mouse IgG (H+L), F(ab’)2 Fragment (Alexa Fluor^®^ 488 Conjugate, 4408S, Cell Signaling Technology, Boston, MA, USA). All steps following this required protection from light. After incubating at room temperature for 1 h, the sections were washed three times and mounted using an anti-fade mounting medium containing DAPI (Beyotime Biotechnology, Shanghai, China). It was important to ensure the removal of air bubbles. The sections were subsequently sealed with nail polish. Finally, the samples were observed and imaged using a confocal microscope.

### 2.4. Tissue Clearing and 3D Staining

In this study, 3D imaging includes both confocal and light-sheet microscopy-based 3D imaging. The procedures for confocal microscopy included tissue clearing, 3D staining, refractive index (RI) matching, and 3D imaging. For tissue clearing, a faster clearing speed of hydrophobic methods (Supplementary Table S3) was performed using a tissue clearing kit (Cat# 240920-12, Nuohai Life Science Co., Ltd., Shanghai, China) [16]. Lipid removal was carried out by immersing the tissue in a lipid removal reagent and incubating it at room temperature with gentle shaking at 60 RPM for 24 h.

For 3D staining, after washing the samples three times with PBS, they were incubated with the same primary antibodies used for 2D sections. Following 24 h of incubation with the primary antibodies, the samples were washed three times and then incubated with secondary antibodies. The volume of the staining solution was maintained at ten times the volume of the sample, and the antibody dilution used for 3D staining was double that of the 2D staining protocol. For refractive index matching, after 24 h of incubation with secondary antibodies, the samples were washed three times in PBST and then transferred into an RI matching solution with a refractive index of 1.51 at room temperature for 2 h. (Figure 1C).

For tissue samples prepared for 3D imaging using light sheet microscopy, the tissue clearing, staining, and refractive index matching followed the same protocol as for confocal imaging, with longer processing times due to the larger sample volume (Table 1). Prior to imaging, the tissue needed to be embedded. Specifically, 1% agarose (agarose/RI matching solution) was heated to 75 °C until fully dissolved (approximately 20 min), then transferred to a 45 °C water bath for 1 h to remove bubbles. After the bubbles were eliminated, a portion of the agarose solution was added to a mold and placed at 4 °C for 20 min to reach a semi-solid state. The tissue sample was positioned in the agarose, and the remaining solution was added to immerse the sample. The mold was then placed at 4 °C for at least 6 h to allow the agarose to solidify completely. All steps were performed at 4 °C to prevent photobleaching of WGA fluorescence, except for refractive index matching, as the index-matching solution may crystallize at this temperature.

### 2.5. 3D Imaging

For confocal 3D staining, samples that have undergone refractive index matching are placed in a confocal dish, with the tissues submerged in the matching solution. Observations are made using a 10× or 20× objective lens. First, an appropriate field of view in the target area is selected, and the focus is adjusted to the lowest (or highest) position, which is set as the starting point for the Z-stack. The focal plane is then gradually raised until the opposite end of the intestine is reached, establishing the endpoint for the Z-stack. For each channel, suitable exposure values are selected, and imaging is performed with a fixed slice thickness of 3 µm. Imaging parameters are set as follows: resolution of 1024 × 1024, speed of 200, frame averaging of 2, and magnification of 0.75. Ensure the total imaging depth stays within 500 µm. Exceeding this depth with higher magnifications may cause the objective lens to touch the dish bottom.

For 3D imaging using light sheet microscopy, the agarose embedded tissue is placed in a 15 mL EP tube with immersion oil and shaken at room temperature at a speed of 60 RPM for at least 2 h to achieve refractive index matching. After that, the embedding block is securely fixed onto the imaging stage and inserted into the imaging chamber full of immersion oil. A tiling light sheet microscopy (Nuohai LS18, Nuohai Life Science Co., Ltd., Shanghai, China) [20] with an objective lens of either 4× or 6.3× magnification is selected, the light sheet is set up to lay flat four to six times depending on the magnification chosen. The position is then adjusted to locate the target field of view, and boundaries for the anterior, posterior, lateral, and vertical limits are established to ensure that all tissue is included within the entire setup range. Automatic exposure is selected to avoid variations in exposure levels across different positions, and then imaging is performed. A performance comparison of different 3D imaging modalities is provided in Supplementary Table S4.

### 2.6. Post-Processing

The post-processing of nerve and vascular imaging data was conducted using Imaris software (Imaris 10.1.0, Oxford Instruments, High Wycombe, UK). Specifically, raw data from both confocal microscopy and light-sheet microscopy were first converted into IMS format using the File Converter software (Imaris 10.1.0, Oxford Instruments, High Wycombe, UK). For light-sheet microscopy data, which often involves multiple scanning regions, the Nuohai Combine and Stitch program (Nuohai Life Science Co., Ltd., Shanghai, China) was used to merge these regions before the IMS conversion.

In Imaris, the “Surface” module was employed to segment fluorescence-positive regions and perform 3D reconstructions. In the Surfaces module, fluorescent structures were modeled using “Background Subtraction”. The subtraction level was defined by two parameters—minimum intensity threshold and the diameter of largest sphere that fits into the object—selected based on how well the resulting surface matched the raw fluorescence. Surfaces were then smoothed with proper “surface detail” parameter. Parameter choices were made by one analyst and confirmed by two independent evaluators by consensus. To ensure comparability, these three parameters were kept identical across groups and specimens for each outcome. The exact values are provided in Table 2 as follow:

For 3D ROIs, ROIs were randomly placed within predefined anatomical compartments while keeping physical size identical across groups.

Quantitative data such as fluorescence intensity and the total volume were extracted from the reconstructed models for further analysis. For intensity, comparisons were made under identical acquisition and imaging conditions across all groups. Because both the view size and imaging parameters were matched, these measurements are standardized and directly comparable.

### 2.7. Statistical Analysis

Sample sizes (n = 5 per group) were determined based on the 3Rs principle, feasibility/cost, and statistical considerations. Quantitative data are expressed as mean ± standard deviation values. A priori normality (Shapiro–Wilk test with Q–Q plot) and homoscedasticity (Levene/Brown–Forsythe) were assessed. For normal data with equal variances, we used two-sided Student’s *t*-test; for normal but unequal variances, Welch’s *t*-test were used; and for non-normal data, Wilcoxon rank-sum test were used for comparison. *p* < 0.05 was considered to be statistically significant. The SAS version 9.2 software (SAS Institute, Cary, NC, USA) was used for all statistical analyses. Images were de-identified and randomized by an independent team member. Analysts were blinded to group allocation. When subjective judgment was involved, assessments were independently double-rated by ≥2 evaluators; discrepancies were resolved by consensus, and only consensus outcomes were used.

## 3. Results

### 3.1. Tissue Harvest and Processing

**Figure 2:**
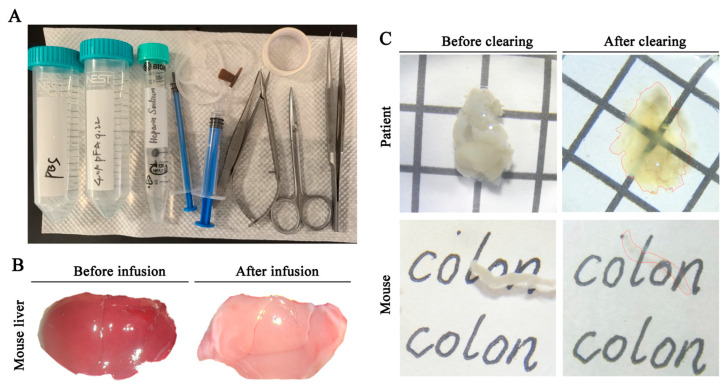
**Overview of tissue fixation, perfusion, and clearing for 3D neurovascular imaging.** (**A**) Supplies required for perfusion fixation, including sodium heparin, phosphate-buffered saline (PBS), 4% paraformaldehyde (PFA), and surgical instruments for tissue harvesting. (**B**) Transition of the liver from red to white during perfusion, indicating successful flushing of red blood cells from the vasculature. (**C**) Comparison of mouse and human intestinal tissues before and after optical clearing. Red dashed lines outline the contours of the cleared tissues for better visualization.

Effective tissue fixation and transparency enhancement are essential for high-quality imaging of neurovascular structures. Figure 2A showed the necessary supplies for perfusion fixation, including sodium heparin, PBS, PFA, and the surgical instruments required for tissue harvest. Figure 2B illustrated the transition of the liver from red to white after perfusion, as red blood cells were flushed out of the vasculature. This facilitated subsequent tissue clearing. As shown in Figure 2C, the colon achieved excellent transparency following optical clearing, allowing light to penetrate smoothly without scattering.

### 3.2. Comparison of Advantages and Disadvantages of Different Imaging Methods

Table 1 provided a detailed comparison of three imaging techniques utilized for visualizing the vascular system. Among these, confocal 3D imaging exhibited notable advantages, including reduced total workflow time (from tissue harvesting to staining and imaging), simpler operational steps, and enhanced capability for 3D reconstruction, post-processing, and quantification, compared to alternative imaging methods. Notably, confocal 2D imaging achieved the highest magnification, whereas light-sheet microscopy provided the most extensive imaging field of view.

### 3.3. Comparison of Different Staining Methods in Imaging the Nerve Fibers

**Figure 3:**
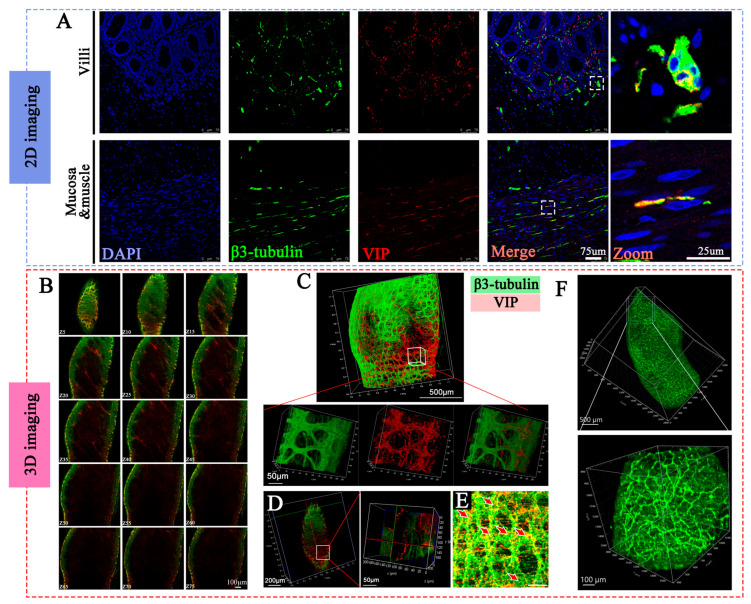
**Comparison of 2D and 3D imaging of colonic nerves.** (**A**) 2D staining and imaging of colonic tissues, showing both the villus and muscular layers with magnified views of local nerve fibers. (**B**) Confocal 3D staining, displaying 2D images at different tissue depths. (**C**) Three-dimensional visualization of colonic nerve fibers, clearly demonstrating longitudinal and transverse neurons. Magnified views highlight distinct nerve channels. (**D**) Cross-sectional views of 3D nerve staining with corresponding magnified regions. (**E**) Localized staining of colonic nerves, showing distinct clusters of ganglia (red arrows). (**F**) Light-sheet 3D staining of nerve fibers, revealing the overall distribution and magnified views. Notably, light-sheet imaging accommodates the largest sample size but with lower imaging resolution. Green: β3-tubulin, marking all nerve fibers; Red: VIP, marking VIP+ neurons. Blue: DAPI staining.

Figure 3A displayed the imaging results from 2D section staining of neurons in colon tissue. Two neural fiber markers, β3-tubulin (Tuj1), and VIP, were utilized to label the enteric nerves. The images revealed a dense network of nerve fibers within both the mucosal layer and the interstitial space. However, it is hard to present the intact structure of the nerve. Figure 3B–D presented the results of confocal 3D staining of the intestinal tissue. In contrast to the 2D sections, confocal imaging allows for clear visualization of the intestinal tissue at various heights (Figure 3B). Figure 3C,D clearly revealed the longitudinally oriented thick intermuscular nerve trunks and the transversely oriented fine submucosal nerve plexuses intertwined within the intestinal tissue, and the magnification showed different channels and different planes of the local region. Additionally, clusters of ganglion cells were observed within the inter-muscular nerve plexus (Figure 3E, red arrow), collectively forming a complex enteric nervous network. The Figure 3F presented the results from light sheet microscopy 3D imaging, which captured the overall distribution of nerve fibers throughout the entire intestinal structure, along with localized magnification of specific areas. However, the imaging quality obtained through light sheet microscopy was not as high as that achieved with section staining and confocal 3D imaging.

### 3.4. Comparison of Different Staining Methods in Imaging the Vasculature

**Figure 4:**
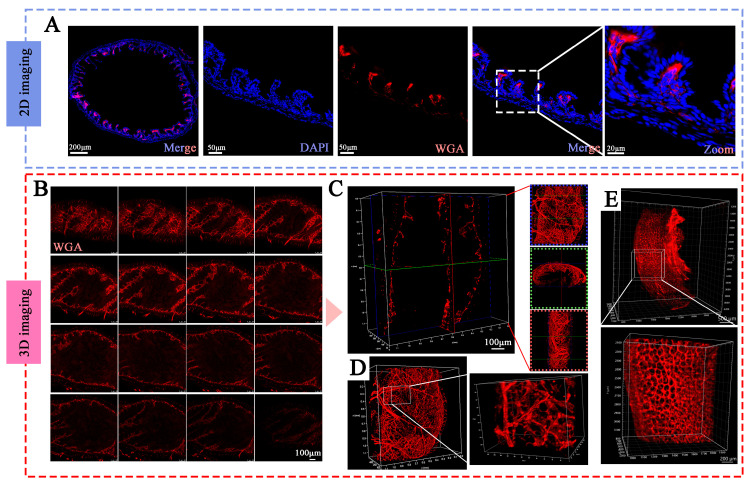
**Comparison of 2D and 3D imaging of colonic blood vessels.** (**A**) 2D staining and imaging of colonic tissues with magnified views of local blood vessels. (**B**) Confocal 3D staining, displaying 2D images at different tissue depths. (**C**) Three-dimensional visualization of colonic blood vessels, illustrating vascular trajectories and network distribution from coronal, transverse, and sagittal views. (**D**) Overall 3D visualization with magnified local regions. (**E**) Light-sheet 3D staining of colonic blood vessels, showing the overall vascular distribution and magnified views. Red: WGA, a dye perfused through the vasculature that binds to the endothelial cell surface. Blue: DAPI staining.

Figure 4A presented the imaging results from 2D section staining of normal mouse colon tissue, along with an enlarged view of a specific area. The sections revealed a dense network of blood vessels arranged in both longitudinal and transverse orientations. In Figure 4B–D, the results of confocal 3D staining of the same intestinal tissue were shown. This imaging method provided high-quality visualization of blood vessels at various heights within the tissue (Figure 4B). Different planes of the WGA signal clearly depicted an intact vascular tree of the intestine along with local magnification (Figure 4C,D).

Figure 3E displayed the results from light sheet microscopy 3D imaging, which illustrated the entire distribution of blood vessels throughout the intestinal structure, along with localized magnified images. While the imaging sample was evidently longer than that of confocal 3D imaging, the quality obtained through light sheet microscopy was inferior to that achieved with 2D section and confocal 3D imaging.

To provide a more intuitive visualization of the 3D structure of nerves and blood vessels, we also created a 3D imaging video of the neurovascular system, as shown in Supplementary Video S1.

### 3.5. Applications of 3D Imaging

Abnormalities in intestinal neurovascular structures were among the most common manifestations of congenital diseases. In HSCR, improper migration of neural crest cells results in the intestine lacking normal relaxation movements, leading to a series of life-threatening clinical conditions. 3D staining provides an excellent tool for investigating the phenotypic features associated with HSCR.

**Figure 5:**
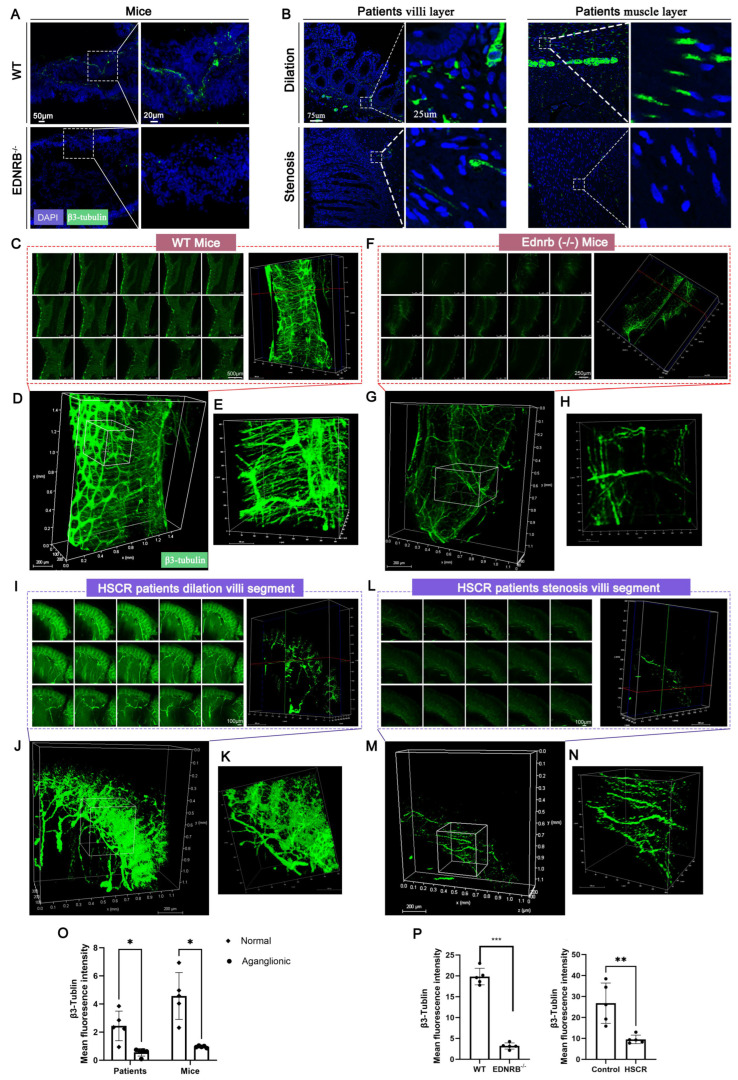
**Application of 2D and 3D nerve imaging in HSCR phenotype studies.** (**A**) 2D staining and imaging of colonic nerves in normal and HSCR model mice (*Ednrb*^−/−^), with magnified views of local nerve fibers. (**B**) 2D staining and imaging of nerves in human normal and HSCR colonic tissues, showing villus and muscular layers with magnified views of local nerve fibers. (**C**–**E**) Confocal 3D staining of normal colon, displaying 2D images at different depths, reconstructed 3D fluorescence images, and magnified views, clearly showing longitudinal and transverse nerve plexuses. (**F**–**H**) Confocal 3D staining of HSCR diseased colon, showing 2D images at different depths, reconstructed 3D fluorescence images, and magnified views, revealing sparse longitudinal myenteric nerve plexuses. (**I**–**K**) Confocal 3D staining of the dilated segment of the HSCR colon, showing 2D images at different depths, reconstructed 3D fluorescence images, and magnified villus layers, with dense villus nerve fibers. (**L**–**N**) Confocal 3D staining of the narrowed segment of the HSCR colon, showing 2D images at different depths, reconstructed 3D fluorescence images, and magnified muscular layers, demonstrating sparse myenteric nerve fibers and absent villus nerve fibers. (**O**) 2D staining shows significantly lower nerve fiber density in HSCR mouse and human diseased tissues compared to normal mouse colon and the dilated segment of HSCR tissues. (**P**) 3D staining reveals significantly reduced average fluorescence intensity of nerve fibers in HSCR transgenic mice and diseased human tissues. Green: β3-tubulin staining. * *p* < 0.05, ** *p* < 0.01, *** *p* < 0.001. n = 5 per group.

Figure 5 compared 2D and 3D staining techniques in analyzing nerve fiber expression in HSCR. Figure 5A,B illustrated the application of 2D staining to monitor nerve fiber expression in normal and HSCR tissues. A significant reduction in nerve fiber expression was observed in diseased tissues (*Ednrb*^−/−^ intestines and the narrowed segment of HSCR patient intestines) compared to their counterparts (WT mice or the dilated segment of HSCR intestines) (Figure 5O). Figure 5C–N showed the 3D staining results of nerve fibers in normal and HSCR tissues from both mice (Figure 5C–H) and humans (Figure 5I–N). Compared to 2D staining, 3D staining offered a clearer and more comprehensive visualization of the 3D structure of nerve fibers. Similarly, in *Ednrb*^−/−^ mice or HSCR narrowed segments, the average fluorescence intensity of nerve fibers was significantly reduced (Figure 5P). These findings suggest that 3D staining is more practical and accurate than traditional 2D imaging for visualizing nerve fibers, providing a robust approach for phenotypic analysis of nerve migration and expression.

**Figure 6:**
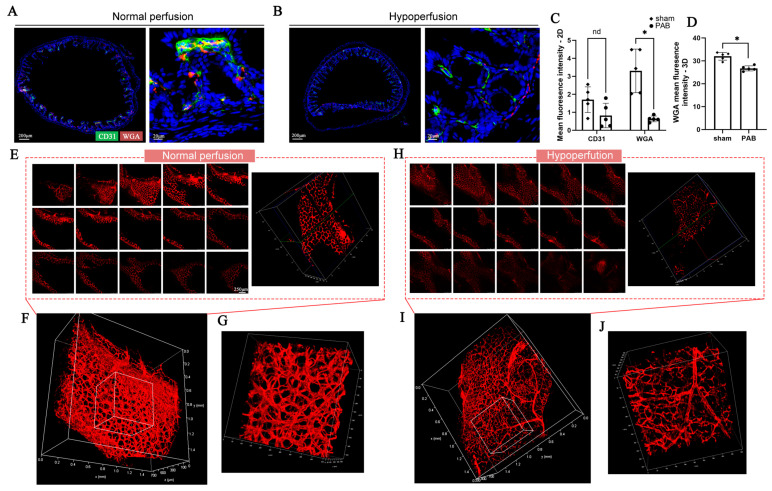
**Application of 2D and 3D vascular imaging in vascular development phenotype studies.** (**A**,**B**) 2D staining and imaging of colonic blood vessels in normal and reduced systemic perfusion groups, with magnified views of vascular-rich regions. (**C**) Quantification of average fluorescence intensity for CD31 and WGA in normal and systemic hypoperfusion groups (PAB group), showing an evident decrease in the PAB group. (**D**) Quantification of spatial average fluorescence intensity of WGA in normal and PAB groups, demonstrating a significant reduction in the PAB group. (**E**–**G**) Confocal 3D staining of normal colon, displaying 2D images at different depths, reconstructed 3D fluorescence images and magnified views, showing dense vascular networks. (**H**–**J**) Confocal 3D staining of colonic tissue from the PAB group, displaying 2D images at different depths, reconstructed 3D fluorescence images, and magnified views of vascular structures. Red: WGA staining; Green: CD31 staining; Blue: DAPI staining. * *p* < 0.05, nd: no significant difference. n = 5 per group.

Right heart system diseases constitute a substantial portion of congenital heart disease; the right ventricular outflow tract obstruction (RVOTO) leads to decreased systemic perfusion, adversely affecting pulmonary vascular development [17,18]. However, there is limited research on how neonatal RVOTO impacts intestinal vascular development. In this study, we established a PAB model in neonatal mice and successfully reduced systemic blood perfusion. We then harvested colon tissues for 2D and 3D vascular imaging (Figure 6). Figure 6A,B presented the 2D staining results of colonic blood vessels under normal and low perfusion conditions. In vascular imaging, CD31 and WGA specifically bind to endothelial cells, clearly outlining the cross-sectional profile of blood vessels (Supplementary Figure S1A,B). Both the specificity and sensitivity of WGA perfusion were greater than 90% (Supplementary Figure S1C). As shown in Figure 6C, reduced blood perfusion was associated with a significant decrease in vascular fluorescence intensity, along with a downward trend in CD31-positive endothelial cells.

Figure 6E–J illustrated the 3D morphology of blood vessels under normal (Figure 6E–G) and hypoperfusion (Figure 6H–J) conditions. Compared to 2D staining, 3D imaging not only preserved the integrity of the tissue but also revealed the vascular branching network within the colon. Similarly, we observed that congenital reductions in blood perfusion were accompanied by a significant decrease in vascular fluorescence intensity (Figure 6D). These findings suggested that early pulmonary hypoperfusion can adversely affect intestinal vascular development, revealing the vascular phenotypes associated with congenital heart disease-related intestinal disorders.

### 3.6. Post-Processing of 3D Imaging

In the post-processing workflow, images were segmented and reconstructed to visualize the spatial organization of nerve fibers. The 3D rendering clearly illustrated the trajectory of the neurovascular structures, facilitating the identification of their variations and spatial relationships. Additionally, by tracking and localizing neurovascular structures, various quantitative metrics can be obtained, including the volume and lengths of individual or total neurovascular networks.

**Figure 7:**
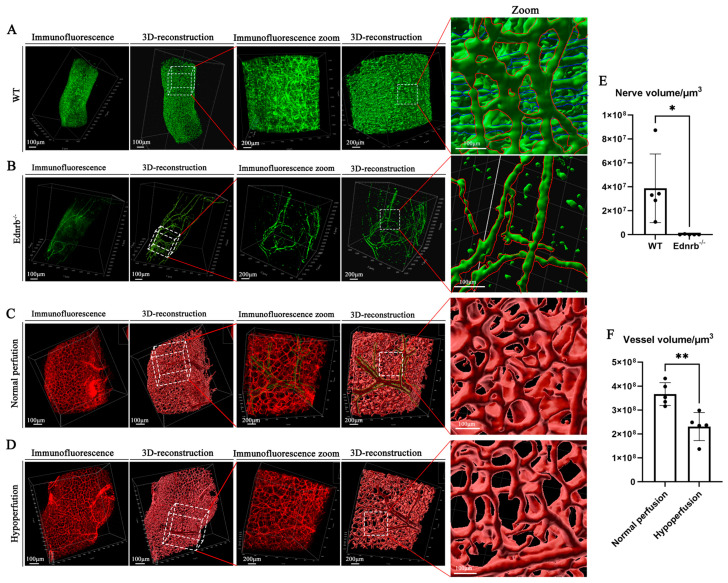
**Post-processing, 3D reconstruction, and application of neurovascular imaging in phenotype studies.** (**A**) 3D staining and reconstruction of nerve fibers in normal colon, with magnified fluorescence and 3D models showing longitudinal (red outline) and transverse (blue outline) nerve plexuses. (**B**) 3D staining and reconstruction of nerve fibers in *Ednrb*^−/−^ colon, revealing sparse nerve structures. (**C**) 3D staining and reconstruction of blood vessels in normal colon, with magnified fluorescence and 3D models showing dense vascular networks, including fine vascular signals outlined in green. (**D**) 3D staining and reconstruction of blood vessels in colonic tissue with reduced systemic perfusion, showing relatively sparse vascular networks. (**E**) Quantification of total nerve volume in 3D reconstructions of normal and HSCR diseased segments. (**F**) Quantification of vascular tree volume in normal and reduced systemic perfusion groups at P10. Red: WGA staining; Green: VIP staining. * *p* < 0.05, ** *p* < 0.01. n = 5 per group.

As shown in Figure 7A,B, we reconstructed the enteric nerve fiber distribution in normal and HSCR (*Ednrb*^−/−^) tissues. The reconstructed images were consistent with the original fluorescence results, and the enlarged local views clearly demonstrated the distribution characteristics of nerve fibers, including the longitudinal myenteric plexus (Figure 7A, red outline) and the transverse submucosal plexus (Figure 7A, blue outline). In contrast, nerve fibers in HSCR tissues were significantly reduced, with only sparse longitudinal plexuses remaining. Quantitative analysis revealed that the density and volume of nerve fibers in HSCR tissues were markedly lower than those in normal tissues (Figure 7E).

Furthermore, we imaged and analyzed the vascular networks of colonic tissues with normal and congenital systemic hypoperfusion (Figure 7C,D). Through 3D reconstruction, we were able to distinguish vascular morphologies that are otherwise difficult to discern with the naked eye due to the dense overlap of fluorescence signals (Figure 7D, green dashed line), thereby improving the accuracy of quantification. Subsequent quantitative analysis indicated that in cases of congenital systemic hypoperfusion, the density and volume of the intestinal vascular network were significantly lower than those in the normal perfusion group (Figure 7F).

To visualize the 3D structure of post-processed blood vessels, we also created a 3D imaging video, as shown in Supplementary Video S2. For larger clinical tissues, we used light sheet microscopy to image and displayed the spatial distribution and reconstruction of nerves, as shown in Supplementary Video S3.

## 4. Discussion

The aim of this study was to develop a novel, efficient, and detailed protocol for tissue clearing, neurovascular staining, and 3D imaging. We systematically compared three imaging techniques—2D imaging, 3D confocal microscopy imaging, and 3D light-sheet microscopy imaging—in terms of six critical parameters: total workflow time, imaging quality, imaging range, operational complexity, post-processing, and quantitative analysis. These methods were subsequently applied to investigate the phenotypes of congenital neurovascular abnormalities.

Our results demonstrated that 3D confocal imaging performed well across multiple criteria, making it a useful approach for detailed phenotypic studies of colonic blood vessels and nerves. However, when specific requirements such as subcellular structural resolution or large-scale imaging of entire organs are needed, 2D imaging and light-sheet microscopy exhibit clear advantages over confocal 3D imaging.

While previous studies have also reported successful neurovascular 3D staining and with effective tissue clearing and imaging [21,22], our method introduced a more simplified, and time-efficient clearing protocol. Notably, when applied to mouse distal colon tissue, the entire procedure can be completed within four days to produce high-quality 3D structures—or even within a single day when autofluorescence is present. Additionally, our comparison between confocal and light-sheet microscopy highlighted their respective strengths and limitations. It is important to select appropriate imaging approaches based on specific research objectives and experimental requirements.

A significant advantage of 3D imaging lies in its application to lineage tracing studies, such as neurons or glial cells, vascular endothelial cells, or smooth muscle cells [23,24]. The most critical aspect of lineage tracing is the accurate visualizing of the spatial distribution of the target cells, which is challenging to achieve with traditional 2D imaging methods. Additionally, our tissue clearing and staining method effectively preserves endogenous fluorescence, making it particularly well-suited for lineage tracing studies. Ongoing work is focused on adapting and promoting this method for lineage tracing study.

Additionally, with advancements in 3D printing technology, the integration of 3D imaging and 3D printing allows for personalized 3D reconstruction and intuitive analysis, which may contribute to the advancement of precision medicine. In this context, our current 3D staining and imaging techniques could serve as a new platform for such integration.

Our methods also present certain limitations. First of all, achieving complete tissue transparency remains a challenge, leading to reduced light penetration and increased blurriness in regions farther from the objective lens. This issue can be mitigated by extending the tissue delipidation time during the clearing process or increasing the refractive index matching time. Secondly, the human cohort size (n = 5) was limited; validation in larger, multi-center cohorts with harmonized pre-analytical variables will be important in the future study. Thirdly, intraoperative ischemia time was not prospectively recorded for the HSCR specimens. Because prolonged ischemia may lead to blood stasis/clotting, which can reduce tissue transparency, this may introduce variability. However, to minimize pre-analytical variation, all clinical comparisons were performed on patient-matched tissues (aganglionic versus ganglionic segments from the same individual), and specimens were handed off in the operating room and fixed immediately upon receipt. Finally, a broader ENS marker panel would further strengthen clinical interpretability of 3D phenotyping. Based on the prior literature, SOX10 and S100B are suitable complementary markers in the HSCR context [25,26]. Because whole-mount 3D staining requires additional validation for antibody penetration and specificity across tissue depth, we did not include these markers in the current study; we will validate SOX10/S100 (and related ENS markers) in future work.

## 5. Conclusions

In this study, we present a rapid and accessible workflow that yields consistent 3D visualization of colonic neurovascular structures. By comparing 2D and 3D imaging approaches and illustrating their potential applications in intestinal neurovascular phenotyping, we found that confocal 3D staining showed generally good performance across multiple evaluation criteria. Accordingly, we suggest confocal 3D staining as a practical option for routine neurovascular phenotyping, while the optimal choice should ultimately depend on the specific experimental goals and imaging requirements.

## Figures and Tables

**Table 1:** Comparison of the advantages and disadvantages of different imaging methods.

		Confocal 2D	Confocal 3D	Light Sheet 3D
**Image** **Quality**	**Pixel/Voxel**	**1024 × 1024**	**1024 × 1024 × 150**	**1024 × 1024 × 1024**
	**Magnification**	**20×–63×**	**10×–20×**	**0.63×–6.3×**
**Workflow**		Fixation → Dehydration → Embedding and sectioning → Deparaffinization → rehydration → Staining, and mounting	Fixation → Clearing → Staining and index matching	Fixation → Clearing → Staining → Agarose embedding → Index matching
**Workflow time**		5.95 ± 0.72 days	4.65 ± 0.71 days	7.95 ± 0.86 days
**Imaging range**		20×: 581 × 581 µm 63×: 184 × 184 µm	20×: 581 × 581 × 700 µm 10×: 1162 × 1162 × 700 µm	Max: 2.6 × 10 × 2.6 cm
**Post-processing**		None	Segmentation, Stitching, Deconvolution, Filaments, Animation	
**Quantification**		Cross-sectional area; Mean fluorescence density	Mean fluorescence density; Volume; Length; Morphology and branching patterns	

**Table 2:** Imaris surface segmentation parameters used for 3D quantification across markers and species.

Parameter	Mice Vip	Mice Tuj1	Human Tuj1	Mice WGA
**minimum intensity threshold**	6.2	2.27	2	1.74
**diameter of largest sphere that fits into the object**	15	8.52	7.5	1
**surface detail**	10.6	8.39	6.5	1.3

## Data Availability

All data and materials used or analyzed during the current study is available from the corresponding author upon reasonable request.

## Supplementary Materials

The following supporting information can be downloaded at: https://www.mdpi.com/article/10.3390/diagnostics16050759/s1, Supplementary Video S1: Confocal 3D imaging of neurovascular structures in mouse intestinal tissue. Supplementary Video S2: Dynamic 3D imaging and modeling of neurovascular structures. Supplementary Video S3: 3D visualization of nerve in human HSCR stenosed and dilated intestinal segments imaged by light sheet microscopy. Supplementary Table S1. The detailed clinical information of Human samples. Supplementary Table S2. Human cohort characteristics across imaging modalities. Supplementary Table S3. Comparison of commonly used tissue-clearing methods with our approach. Supplementary Table S4. Comparison of commonly used 3D imaging modalities. Supplementary Figure S1 Assessment of WGA perfusion sensitivity and specificity. References [11,12,13,27,28,29,30,31,32,33,34,35,36] are cited in the supplementary materials.

## Abbreviations

2/3D	2/3-dimensional
HSCR	Hirschsprung’s disease
NEC	Necrotizing enterocolitis
β3-tubulin	Neuron-specific class III beta-tubulin
VIP	Vasoactive intestinal peptide
WGA	Wheat germ agglutinin
PAB	Pulmonary artery banding
PBS	Phosphate-buffered saline
PFA	Paraformaldehyde
RI	Refractive index
