# Supplementary material for: A Novel Rapid 3D Tissue-Clearing and Staining Approach for Enteric Neurovascular Imaging and Pathology Applications
Source: Diagnostics (Basel). 2026 Mar 3;16(5):759. doi: 10.3390/diagnostics16050759 (PMC12984641; doi:10.3390/diagnostics16050759)
Supplement: Supplementary file 1 [file diagnostics-16-00759-s001.zip › Supplemental Figure 1+Supplemental Table 1-4.pdf]

A Novel Rapid 3D Tissue-Clearing and Staining Approach for Enteric Neurovascular Imaging and Pathology Applications

Debao Li [1] , Xuqing Cao [1] , Jienan Lin [2] , Qingchi Zhang [3], Rui Dong [1] , Song Sun [1,*] , Chun Shen [1,*]
1.National Health Commission (NHC) Key Laboratory of Neonatal Diseases, Department of Pediatric Surgery, Children's Hospital of Fudan University, Shanghai 201102, China; lidebaoemail888@163.com (D.L.); 22111240002@m.fudan.edu.cn (X.C.); rdong@fudan.edu.cn (R.D.)
2.Department of Neonatal Surgery, The Affiliated Women and Children' s Hospital of Ningbo University, Ningbo 315012, China; felinjienan@nbu.edu.cn
3.Xiamen Key Laboratory of Pediatric General Surgery Diseases, Children's Hospital of Fudan University (Xiamen Branch), Xiamen Children's Hospital, Xiamen, 361006, China; zqc1403@126.com
*Correspondence: sun_song@fudan.edu.cn (S.S.); chunshen0521@126.com (C.S.);
Tel.: +86-021-64931212 (S.S. & C.S.)

Supplementary Figure S1 Assessment of WGA perfusion sensitivity and specificity.

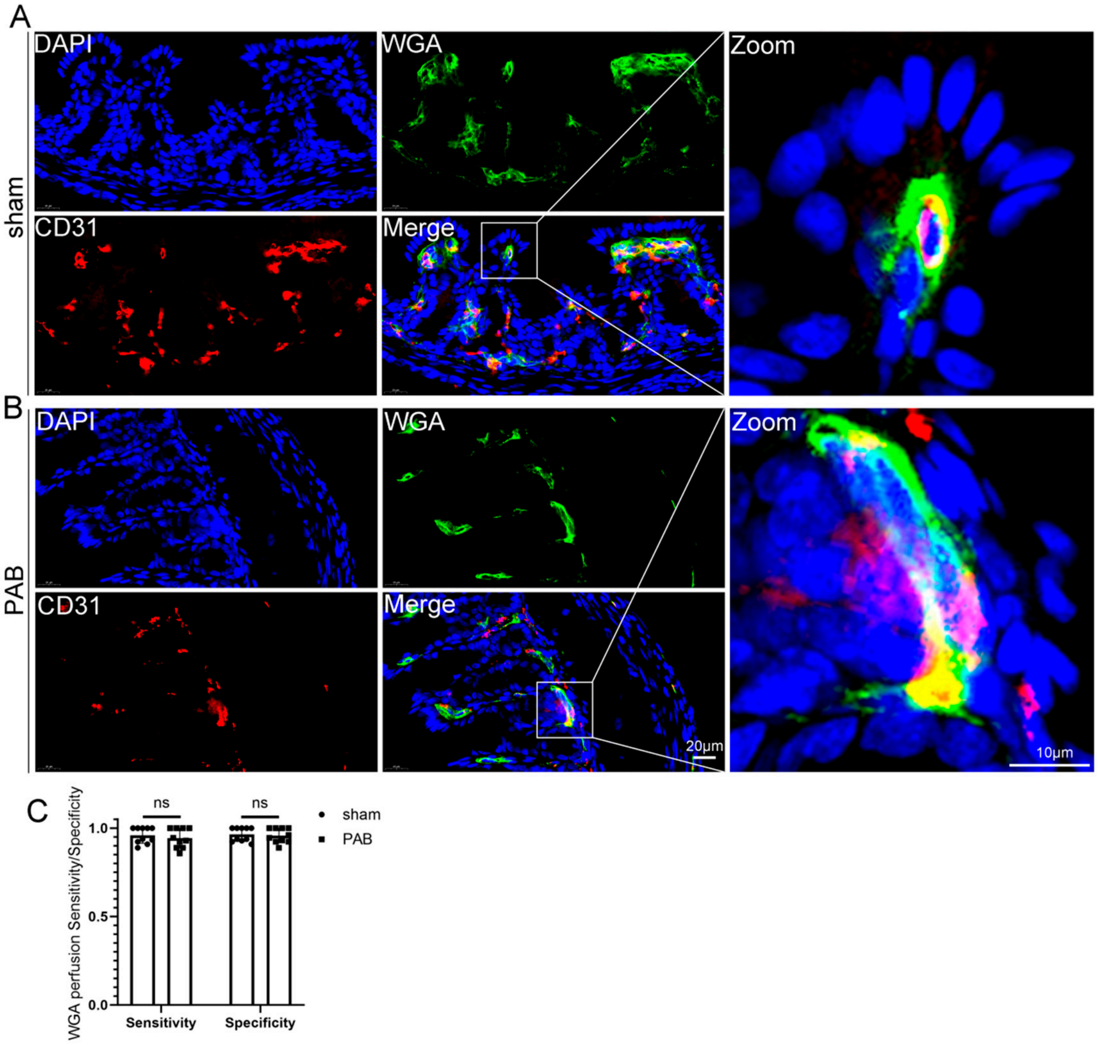

A, Representative co-staining images of WGA (green) and the endothelial marker CD31 (red) in the sham group, with corresponding zoomed-in views.

B, Representative co-staining images of WGA (green) and CD31 (red) in the PAB group, with

corresponding zoomed-in views.

C, Quantification of WGA perfusion sensitivity and specificity.Scale bars: 20 μm (main images) and 10 μm (zoomed images). ns means No significance.

Supplemental Table S1. The detailed clinical information of Human samples

Subject.name	Diagnosis	Classification of UpToDate	Classification of Chinese Guideline	Segment	Age	Sex	Sample Region	Sample Position （Centre Biopsy Point）	Usage
Subject_1	Hirschsprung disease	Long	Long	Normal	11M3D	Female	Colon	40 cm proximal to the anus	Confocol 2D
Subject_1	Hirschsprung disease	Long	Long	Aganglionic	11M3D	Female	Colon	10 cm proximal to the anus	Confocol 2D
Subject_2	Hirschsprung disease	Short	Short	Normal	3M10D	Male	Colon	20 cm proximal to the anus	Confocol 2D
Subject_2	Hirschsprung disease	Short	Short	Aganglionic	3M10D	Male	Rectum	8 cm proximal to the anus	Confocol 2D
Subject_3	Hirschsprung disease	Short	Short	Normal	8M21D	Male	Colon	35 cm proximal to the anus	Confocol 2D
Subject_3	Hirschsprung disease	Short	Short	Aganglionic	8M21D	Male	Rectum	10 cm proximal to the anus	Confocol 2D
Subject_4	Hirschsprung disease	Short	Common	Normal	1Y4M	Male	Colon	Proximal sigmoid colon(the exact length of the ganglionic bowel of this subject was not measured during surgery)	Confocol 2D
Subject_4	Hirschsprung disease	Short	Common	Aganglionic	1Y4M	Male	Rectum	5 cm proximal to the anus	Confocol 2D
Subject_5	Hirschsprung disease	Short	Common	Normal	3M8D	Female	Colon	3cm proximal to anus	Light sheet 3D
Subject_5	Hirschsprung disease	Short	Common	Aganglionic	3M8D	Female	Colon	20cm proximal to anus	Light sheet 3D
Subject_6	Hirschsprung disease	Short	Common	Normal	1Y8M	Male	Colon	3 proximal to anus	Confocol 3D
Subject_6	Hirschsprung disease	Short	Common	Aganglionic	1Y8M	Male	Colon	20cm proximal to anus	Confocol 3D
Subject_7	Hirschsprung disease	Short	Common	Normal	1Y8M	Male	Colon	5 cm proximal to anus	Confocol 3D
Subject_7	Hirschsprung disease	Short	Common	Aganglionic	1Y8M	Male	Colon	25cm proximal to anus	Confocol 3D
Subject_8	Hirschsprung disease	Short	Short	Normal	9M13D	Male	Colon	3.5cm proximal to anal dentate line	Confocol 3D
Subject_8	Hirschsprung disease	Short	Short	Aganglionic	9M13D	Male	Colon	20cm proximal to anal dentate line	Confocol 3D
Subject_9	Hirschsprung disease	Short	Common	Normal	1Y9M	Male	Colon	3.5cm proximal to anus	Confocol 3D
Subject_9	Hirschsprung disease	Short	Common	Aganglionic	1Y9M	Male	Colon	20cm proximal to anus	Confocol 3D
Subject_10	Hirschsprung disease	Short	Short	Normal	7M7D	Male	Colon	4cm proximal to anus	Confocol 3D
Subject_10	Hirschsprung disease	Short	Short	Aganglionic	7M7D	Male	Colon	20cm proximal to anus	Confocol 3D
Subject_11	Hirschsprung disease	Short	Common	Normal	6M	Female	Colon	3cm proximal to anal dentate line	Light sheet 3D
Subject_11	Hirschsprung disease	Short	Common	Aganglionic	6M	Female	Colon	20cm proximal to anal dentate line	Light sheet 3D
Subject_12	Hirschsprung disease	Short	Common	Normal	3M17D	Male	Colon	3.5cm proximal to anal dentate line	Light sheet 3D
Subject_12	Hirschsprung disease	Short	Common	Aganglionic	3M17D	Male	Colon	20cm proximal to anal dentate line	Light sheet 3D
Subject_13	Hirschsprung disease	Short	Short	Normal	2M27D	Male	Colon	5cm proximal to anus	Light sheet 3D
Subject_13	Hirschsprung disease	Short	Short	Aganglionic	2M27D	Male	Colon	20cm proximal to anus	Light sheet 3D
Subject_14	Hirschsprung disease	Short	Common	Normal	2M6D	Male	Colon	3cm proximal to anus	Light sheet 3D
Subject_14	Hirschsprung disease	Short	Common	Aganglionic	2M6D	Male	Colon	20cm proximal to anus	Light sheet 3D
Subject_15	Hirschsprung disease	Total	Total	Normal	3M4D	Male	Ileum	25 cm proximal to cecum	Confocol 2D
Subject_15	Hirschsprung disease	Total	Total	Aganglionic	3M4D	Male	Ileum	5 cm proximal to cecum	Confocol 2D

Supplementary Table S2. Human cohort characteristics across imaging modalities

		Confocol 2D	Confocol 3D	Light sheet 3D
**n**		5	5	5
**Age (months)**		8.4±5.1	15.5±6.2	3.6±1.4
**Age (months), median (range)**		8.7 (3.1–16.0)	20.0 (7.2–21.0)	3.3 (2.2–6.0)
**Sex**				
	Male	4	5	3
	Female	1	0	2
**Classification (Chinese guideline)**				
	Short	2	3	4
	Common	1	2	1
	Long	1	0	0
	Total	1	0	0

Supplementary Table S3. Comparison of commonly used tissue-clearing methods with our approach

Dimension	3DISCO / iDISCO / uDISCO / vDISCO, ECi / BABB	CLARITY / PACT / ePACT / SHIELD	CUBIC / Scale / SeeDB / Fast 3D Clear / F-CUBIC	Our method
**Type**	Solvent-based (hydrophobic) method	Hydrogel-embedding method	Aqueous (hydrophilic) method	Hybrid (solvent delipidation with aqueous RI matching)
**Steps**	**Moderate:** dehydration (MeOH/THF) → delipidation (DCM) → high-RI matching (DBE/BABB/ECi)	**Complex:** hydrogel embedding → SDS delipidation (passive/electrophoretic) → RI matching; requires dedicated equipment	**Simple:** immersion delipidation→decolorization/decalsification (optional) → RI matching	**Simple:** Delipidation → RI matching
**Transparency**	**High**	**Medium–high:** stable for large soft tissues	**Medium–high:** stable for soft tissues (brain, gut);	**High**
**Clearing speed**	**Hours to days**	**Days to weeks**	**Hours to days**	**Hours to days**
**Fluorescence preservation**	**Bad:** Endogenous fluorescent proteins quenching; antibody amplification needed	**Good:** excellent protein/epitope retention, compatible with multiple rounds of IHC	**Good**	**Good:** persist for months
**Toxicity**	**High:** THF, DCM, DBE,BABB(volatile/flammable/toxic)	**Moderate:** acrylamide (neurotoxic/possible carcinogen), SDS (irritant)	**Low–moderate:** amines/urea/sugars/alcohols(irritant/corrosive risk)	**Low:** without highly hazardous components
**Ref.**	1.Ueda et al. [27]<br>2.Molbay et al. [28]<br>3.Kosmidis et al. [29]	1.Ueda et al. [27]<br>2.Weiss et al. [30]<br>3.Gradinaru et al. [31]	1.Ueda et al. [27]<br>2.Liu et al. [32]<br>3.Tainaka et al. [33]	

Supplementary Table S4. Comparison of commonly used 3D imaging modalities.

Dimension	Confocal Microscopy	Light-Sheet Microscopy (LSFM/SPIM)	Two-Photon Microscopy (2P)
**Resolution**	**Highest:** lateral: 200–250 nm; axial: 500–700 nm	**Lower:** lateral: 300–600 nm; Axial: 1–5 µm	**Moderate:** lateral: 300–500 nm; axial: 800–1500 nm
**imaging depth**	**Millimeter scale**	**Millimeter–centimeter scale**	**Micrometer–millimeter scale**
**Sample suitability**	Sliced sections; Small cleared tissues (<1mm)	Cleared whole organs/long tissue segments	In vivo thick tissue; blood-flow & calcium imaging;
**Ease of use**	Widely available and easy to use	Less widely available; Higher system complexity (alignment /mounting/RI matching)	Least widely available; Higher operator-training requirement
**Cost**	**Moderate**	**Moderate**	**Expensive**
**Data footprint**	**Moderate;** GB-scale	**Large;** TB-scale	**Moderate;** GB-scale
**Ref.**	Chiang et al. [11]<br>Elliott et al. [34]	Daetwyler et al. [12]<br>Delage et al. [13]	Ota et al. [35]<br>Lee et al. [36]
